# Patients with asymptomatic severe mitral regurgitation already have abnormal left ventricular myocardial sarcomeric ultrastructural characteristics

**DOI:** 10.1016/j.xjon.2026.101821

**Published:** 2026-05-04

**Authors:** Yuanjia Zhu, Seung Hyun Lee, Avi Gupta, Catherine A. Wu, Jinsuh Jung, Akshay Venkatesh, Sidarth Ethiraj, Charles J. Stark, Chris Huynh, Matthew D. Vergel, Shin Yajima, Y. Joseph Woo

**Affiliations:** a Department of Cardiothoracic Surgery, Stanford University, Stanford, Calif; b Department of Bioengineering, Stanford University, Stanford, Calif; c Department of Cardiovascular Surgery, Osaka University Graduate School of Medicine, Osaka, Japan

**Keywords:** mitral regurgitation, left ventricular, sarcomeric

## Abstract

**Objective:**

Mitral valve (MV) surgery remains a class I recommendation for patients with symptomatic severe primary mitral regurgitation (PMR). For asymptomatic patients with severe PMR and preserved left ventricular (LV) function without atrial fibrillation, pulmonary hypertension, or significant left atrial enlargement, surgical treatment is not currently recommended. However, the nature and extent of LV myocardial ultrastructural alterations in patients who are asymptomatic remain unclear.

**Methods:**

Twelve patients with severe PMR were enrolled in this study, 8 of whom were symptomatic and 4 asymptomatic. Three donor hearts were used as controls. LV biopsies were obtained and processed for transmission electron microscopy. Transmission electron microscopy images were acquired from 600× to 25,000× magnification. Quantitative analysis was performed using the ImageJ software. Statistical comparisons were made using a linear mixed-effects model to account for repeated measurements within individuals. Post-hoc pairwise comparisons were conducted using Bonferroni correction. Statistical significance was defined as *P* < .05. This study was approved by the institutional review board.

**Results:**

Qualitatively, myofibrillar disarray, reduced cytoplasmic reserve, and mitochondrial derangement were observed in patients with severe PMR, regardless of presence of symptoms, compared with controls. Quantitatively, the sarcomere length was greater in control patients (1.65 ± 0.23 μm) compared with those who were symptomatic (1.31 ± 0.34 μm, *P* = .049). The mitochondria area was also larger in control patients (0.35 ± 0.19 μm^2^) than in patients who were symptomatic (0.27 ± 0.14 μm^2^, *P* = .33). The mitochondria cristae thickness was greater in control patients (0.05 ± 0.01 μm) compared with patients who were symptomatic (0.02 ± 0.003 μm, *P* < .001) and asymptomatic (0.02 ± 0.03 μm, *P* < .001). Similarly, the mitochondrial major-to-minor axis ratio was greater in control patients (1.86 ± 0.93) than in patients who were symptomatic (1.65 ± 0.52, *P* = .044) and asymptomatic (1.50 ± 0.46, *P* = .003).

**Conclusions:**

In patients with severe asymptomatic PMR and preserved LV function without atrial fibrillation, pulmonary hypertension, or significant left atrial enlargement, ultrastructural alterations of the LV myocardium are already evident and comparable with those observed in severe symptomatic PMR. These findings suggest that early surgical intervention in patients who are asymptomatic with severe PMR may help prevent further adverse remodeling and potentially improve clinical outcomes.

**Figure:**
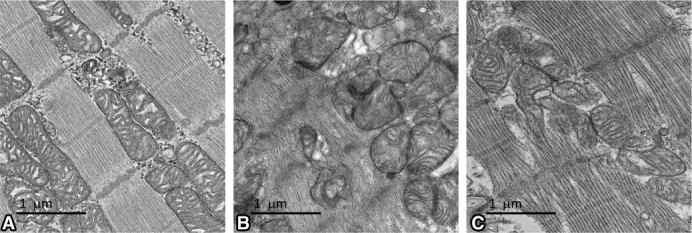
TEM images from control (A), symptomatic (B), and asymptomatic (C) patients.

**Central Message**

In patients with severe asymptomatic primary mitral regurgitation with preserved left ventricular function, ultrastructural damage may exist. Early surgery may prevent adverse remodeling.

**Perspective**

In asymptomatic patients with severe primary mitral regurgitation and preserved left ventricular function, without atrial fibrillation, pulmonary hypertension, or significant left atrial enlargement, surgery is not currently recommended. However, ultrastructural abnormalities may already be present, and early intervention could help prevent adverse remodeling and improve outcomes.

Primary mitral valve regurgitation (PMR) is one of the most common valvular pathologies worldwide.^1^^,^^2^ Currently, surgical intervention with mitral valve (MV) repair or replacement remains a class I recommendation for patients with symptomatic, severe PMR.^3^^,^^4^ In addition, MV repair is the recommended treatment for patients with PMR when the result is expected to be durable.^5^, ^6^, ^7^, ^8^ For patients with severe PMR who are asymptomatic, MV surgery is still unanimously a class I recommendation for those with left ventricular (LV) dysfunction.^3^^,^^4^ The European Society of Cardiology/European Association for Cardio-Thoracic Surgery guidelines further put forward a class I recommendation for MV surgery for those with asymptomatic severe PMR and normal LV function if at least 3 of the following criteria are fulfilled: atrial fibrillation, elevated systolic pulmonary arterial pressure, left atrial (LA) dilatation, and concomitant moderate or greater secondary tricuspid regurgitation.^3^ However, for patients who do not meet the aforementioned criteria, MV surgery remains at most a class II recommendation. It is known that ultrastructural derangements are present in the LV of patients with chronic severe MR.^9^^,^^10^ More importantly, long before the onset of these permanent changes, the LV has already experienced maladaptive remodeling that ultimately fails, leading to progressive LV dysfunction and decompensation.^11^ With advances in modern technologies and significant improvement in MV repair surgical techniques, clinical outcomes have continued to improve.^12^^,^^13^ In this context, earlier surgical intervention for patients with PMR may be reasonable. Currently, surgical intervention in patients with severe PMR and preserved LV function who are asymptomatic, in the absence of other guideline-triggered indications, remains a class II recommendation.^3^^,^^4^ However, data on LV myocardial ultrastructural characteristics in this population are limited. We hypothesized that ultrastructural changes are already present in the LV myocardium of asymptomatic patients with severe PMR who otherwise do not meet class I indication for MV surgery.

## Methods

### Patients

From April 2022 through July 2023, 12 patients with severe PMR who were scheduled to undergo elective MV repair operations were prospectively enrolled in this study. Eight of these patients were symptomatic, and the remaining 4 were asymptomatic with normal LV function without atrial fibrillation, pulmonary hypertension, or LA dilatation. Patient demographic data were collected. In addition, 3 brain-dead donor hearts were randomly selected and included in this study as control patients. These hearts were harvested after diastolic cardioplegic arrest and preserved in SherpaPak, a temperature-controlled hypothermic storage system.^14^ Donor demographic data were collected as well. Average allograft ischemia time before LV biopsy and implantation was 224.0 ± 23.3 minutes. This study was approved by the institutional review board under protocols nos. 32769 and 58850 (approval dates: January 20, 2015, and October 26, 2020). Patients provided informed written consent for the publication of their study data.

### Sample Preparation and Image Acquisition

In patients with PMR, LV biopsies were obtained using biopsy forceps to directly retrieve samples from within the LV cavity through left atriotomy after the heart was arrested. For donor hearts, LV biopsies were obtained immediately before implantation using the same biopsy forceps but via an aortotomy. All biopsies were obtained in the similar area of the LV. Samples were immediately passed off from the field to sterile phosphate-buffered saline (Sigma Aldrich, cat. no. D8537) at 4 °C. Patients with severe PMR continued to undergo elective MV repair operations as per the standard of care. The donor hearts were also used immediately for orthotopic heart transplantation.

The LV biopsies were first divided using a fresh scalpel into 1 mm^3^ in size. These tissue samples were then fixed in a mixture of 2% glutaraldehyde (Electron Microscopy Science, cat. no. 16000) and 4% paraformaldehyde (Electron Microscopy Science, cat. no. 15700) in 0.1 M sodium cacodylate buffer at pH of 7.4 (Electron Microscopy Science, cat. no. 12300) for 1 hour at room temperature. After fixation, the samples were placed in 4 °C fridge for 24 hours. Then, the fixative solution was replaced with cold, aqueous 1% Osmium tetroxide (Electron Microscopy Science, cat. no. 19100) in double-distilled H_2_O (ddH2O), and the samples were allowed to warm to room temperature for 2 hours on a gentle rotator in a hood. The LV samples were then washed 3 times with ddH2O for 5 minutes each, then en bloc stained in 1% uranyl acetate (Fisher Scientific, cat. no. NC1375332) in ddH2O for 2 hours while on the rotator. The fixed LV tissues were dehydrated in 50% and 70% ethanol (EtOH; Sigma-Aldrich, cat. no. 459844) washes for 30 minutes each at room temperature then moved to 4 °C overnight. The samples were then placed in 95% EtOH and allowed to warm to room temperature, then placed in 100% EtOH and washed twice for 10 minutes each, followed by propylene oxide (Thermo Fisher Scientific, cat. no. 149620010) for 15 minutes. Epoxy resin was embedded by using EMbed-812 resin (Electron Microscopy Science, cat. no. 14120) mixed with propylene oxide with a 1:2, 1:1, and 2:1 ratio for 2 hours each. The samples were left in the 2:1 resin to propylene oxide mixture overnight rotating at room temperature in the hood. Lastly, the samples were placed into EMbed-812 for 2 to 4 hours then placed into molds with labels and fresh resin, oriented to allow sectioning along the long-axis of the LV and placed into 65 °C oven overnight.

Ultrathin sections of the LV tissues were taken at 80 nm thick using an UC7 (Leica) with a Diatome Ultra diamond knife, then picked up on formvar/carbon-coated 100 mesh Cu grids (Fisher Scientific, cat. no. 5026044) and stained for 40 seconds in 3.5% uranyl acetate (Electron Microscopy Science, cat. no. 22405) in 50% acetone (Sigma Aldrich, cat. no. 179973). The samples were further stained in Sato's lead citrate (Fischer Scientific, cat. no. 5026892) for 2 minutes. The JEM1400 transmission electron microscope (TEM) 120 kV (JEOL) with a LaB6 emitter was used to acquire images with a Gatan OneView 4k × 4k digital camera from 600× to 25,000× magnifications.

### Data Analysis and Statistics

**Figure 1:**
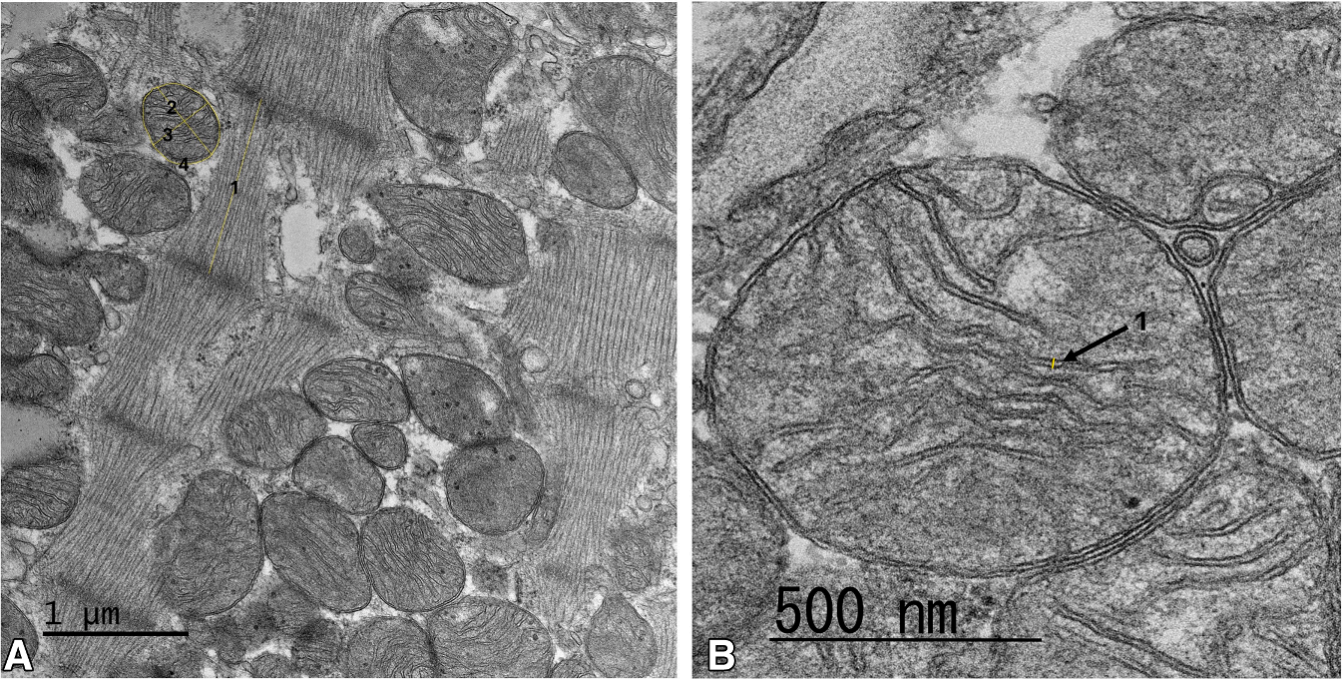
An exemplary transmission electron microscopy image illustrating measurements performed for analysis. Variables include (A) sarcomere length (1), mitochondrial major diameter (2), mitochondrial minor diameter (3), and mitochondrial area (4). B, Mitochondrial cristae thickness (1).

Images captured at 10,000× and 20,000× displacing sarcomeres with visible Z lines and mitochondria were used for quantitative measurements. An exemplary TEM image for analysis is shown in Figure 1. In detail, measurements were performed using ImageJ (National Institutes of Health, version 1.54r). Sarcomere length was measured using the straight-line tool as the distance between Z lines. Mitochondrial area was measured using the freehand selection tool by tracing individual unfragmented mitochondria. Major and minor mitochondrial diameters were measured using line selections across each mitochondrion at the longest and shortest axes. Cristae thickness was measured using perpendicular line selections across cristae membranes. For each variable, 5 measurements were taken on each image. For each patient, at least 2 images were used for analyses. Each measurement was treated as individual observation. All measurements were performed by one observer blinded to patient groups: control versus symptomatic versus asymptomatic. Once all measurements were completed, the patient group was then unblinded for data analysis. Descriptive statistics were presented as raw means and standard deviations of all observations. Statistical comparisons were made using a linear mixed-effects model to account for repeated measurements within individuals. Post-hoc pairwise comparisons were conducted using Bonferroni correction. Statistical significance was defined as *P* < .05.

## Results

### Patient and Donor Demographics

The average age of symptomatic patients with PMR was 68.3 ± 10.8 years, and 4 (50%) were female. Comorbidities included atrial fibrillation in 5 patients (62.5%), pulmonary hypertension in 2 (25%), hypertension in 4 (50%), hyperlipidemia in 3 (37.5%), diabetes in 1 (12.5%), and coronary artery disease in 1 (12.5%). No patients had chronic obstructive pulmonary disease, chronic kidney disease, or prior cerebrovascular accident. Among patients with PMR who were asymptomatic, the average age was 64.5 ± 13.0 years, and all were male. Hypertension was present in 2 patients (50%), hyperlipidemia in 3 (75%), diabetes mellitus in 1 (25%), and coronary artery disease in 1 (25%). The donor group had an average age of 42.0 ± 12.5 years, and 2 (66.7%) were female. No comorbidities were recorded in this group.

### Qualitative Characters

**Figure 2:**
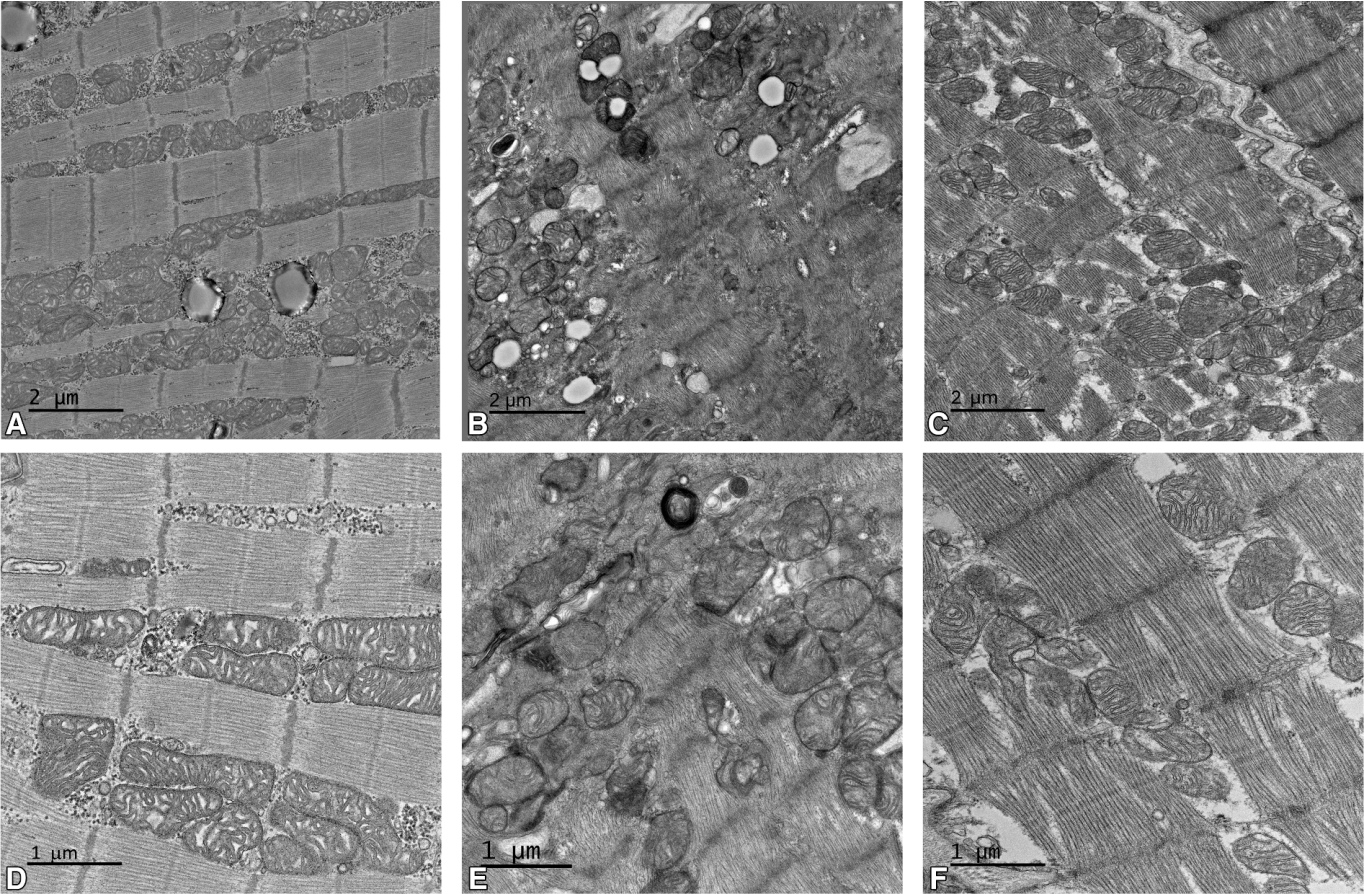
Exemplary transmission electron microscopy images acquired both at low magnification (A-C) and high magnification (D-F). Images were taken from control patients (A and D), patients with symptomatic severe primary mitral regurgitation (*PMR*) (B and E), and patients with asymptomatic severe PMR (C and F).

Qualitatively, LV myocardium from patients with symptomatic severe PMR demonstrated a generally organized myofibrillar structure but with reduced cytoplasmic reserve and interfibrillar spacing. Mitochondrial populations appeared uniform, but disruption of the highly organized linear registry of mitochondria was present. The cytoplasm also contained heterogeneous vesicular and vacuolar structures of varying sizes. LV myocardium from asymptomatic patients with severe PMR exhibited an intermediate phenotype with similarly organized myofibrils with reduced interfibrillar space and cytoplasmic reserve. There was partial preservation of mitochondrial heterogeneity. There were also vacuolar structures observed. Overall, myofibrillar disarray, as well as Z-line thickening and disruption, were observed in patients with severe PMR, regardless of presence of symptoms (Figure 2).

In contrast, LV myocardium from donor control hearts exhibited preserved sarcomeric organization with linear, continuous, and organized myofibrils with clearly identifiable A bands, I bands, and Z lines. There was high mitochondrial volume density, and they were abundant, variably shaped, and organized. Mitochondria were heterogeneous in shape, intact membranes, with intact membranes and well-developed cristae. Numerous prominent clusters of electron-dense granular material were present, consistent with glycogen-rich regions (Figure 2).

### Quantitative Characters

Quantitative measurements of the TEM images of control patients and patients with symptomatic and asymptomatic severe PMR are shown in Tables 1 and 2. Specifically, the sarcomere length was greater in control patients compared with patients who were symptomatic (*P* = .049), but no statistical difference was found when compared with patients who were asymptomatic (*P* = .17). The sarcomere length was similar between patients who were symptomatic and asymptomatic (*P* = 1). The mitochondria area was also different in all groups (*P* = .49). Although statistically significant differences were not observed, the average mitochondria area was larger in control patients than in patients who were symptomatic (*P* = .33). The mitochondria area was similar between control patients and patients who were asymptomatic (*P* = 1). Patients who were symptomatic demonstrated a smaller mitochondria area compared with patients who were asymptomatic (*P* = .069). The mitochondria cristae thickness was greater in control patients compared with patients who were symptomatic (*P* < .001) and asymptomatic (*P* < .001). There was no difference in mitochondria cristae thickness between patients who were symptomatic and asymptomatic (*P* = 1). No difference in mitochondria major diameter was appreciated among the groups. However, the mitochondrial minor diameter was larger in patients who were asymptomatic compared with patients who were symptomatic (*P* = .046), but no difference was observed between the patients who were symptomatic and the control patients (*P* = .41) or between the patients who were asymptomatic and the control patients (*P* = 1). The mitochondrial major-to-minor axis ratio was greater in control patients than in patients who were symptomatic (*P* = .044) and asymptomatic (*P* = .003). The ratio was not statistically different between patients who were symptomatic and asymptomatic (*P* = .08).

**Table 1:** Quantitative measurements of left ventricular myocardial transmission electron microscope images

Group	Sarcomere length, μm	Mitochondria area, μm^2^	Mitochondria cristae thickness, μm	Mitochondria major diameter, μm	Mitochondria minor diameter, μm	Mitochondria major-to-minor axis ratio
Symptomatic	1.31 ± 0.34	0.27 ± 0.14	0.02 ± 0.003	0.70 ± 0.25	0.43 ± 0.13	1.65 ± 0.52
Asymptomatic	1.38 ± 0.25	0.35 ± 0.16	0.02 ± 0.03	0.77 ± 0.24	0.53 ± 0.17	1.50 ± 0.46
Controls	1.65 ± 0.23	0.35 ± 0.19	0.05 ± 0.01	0.83 ± 0.33	0.48 ± 0.14	1.86 ± 0.93
*P* value	.048	.049	<.001	.21	.042	<.001

Analysis was performed using a linear mixed-effects model with post-hoc correction. Statistical significance was defined as *P* < .05.

**Table 2:** Number of observations of each variable of left ventricular myocardial transmission electron microscope images

Group	Sarcomere length	Mitochondria area	Mitochondria cristae thickness	Mitochondria major diameter	Mitochondria minor diameter	Mitochondria major-to-minor axis ratio
Symptomatic	249	349	316	349	349	349
Asymptomatic	90	188	208	188	188	188
Controls	73	70	67	70	70	70

## Discussion

In this study, we observed ultrastructural changes that are already present in the LV myocardium from patients who had asymptomatic severe PMR with preserved LV function without atrial fibrillation, pulmonary hypertension, or significant left atrial enlargement before those patients became symptomatic. In fact, several studies have studied the natural progression of asymptomatic severe PMR. It is known that MR results in volume overload in the LV. The natural compensatory response is myocardial hypertrophy and progressive dilatation to increase the LV end-diastolic volume to diminish wall stress.^11^ The LA also dilates and increases its compliance to maintain normal LA pressure. Therefore, long-standing MR can ultimately result in a decrease in LV function.^11^ The increase in LA and LV end-diastolic pressures can lead to increased pulmonary vascular resistance, causing symptoms of heart failure and clinical decompensation.^15^ However, these pathophysiologic changes are often silent for years before the appearance of functional decline and symptoms.^16^ In response, clinical studies have shown that early surgical intervention for PMR is associated with significant long-term reductions of cardiac-related morbidity and mortality in patients with severe PMR who are asymptomatic.^17^ Without surgical treatment, the annual progression rate from asymptomatic PMR to either symptoms or high-risk LV performance matrices is more than 10%.^18^

To control for any potential confounding factors, LV biopsies for this study were all taken when the heart was in diastolic arrest after cardioplegia, including donor samples. This was to ensure ultrastructural differences attributable to diastolic versus systolic states were minimized.^19^^,^^20^ Under TEM examination, LV myocardial ultrastructural changes were present in patients with severe PMR, regardless of presence of symptoms, when compared with control patients. In control patients, the TEM image findings were consistent with metabolically active donor myocardium and showed no myofibrillar disruption or ultrastructural injury.^21^ However, in patients with symptomatic severe PMR, myofibrillar disarray was prominent, with loss of organized, linear myofibrillar packing. Disruption, lysis, and fragmentation of Z lines were also observed, suggesting myofibrillar lysis. In addition, Z-line thickening and disruption were present, and sarcomere shortening was observed. Similar myofibrillar changes were also observed in patients with severe PMR who were asymptomatic but perhaps to a slightly lesser degree. These findings suggest adaptation changes, and even decompensated remodeling specifically in the symptomatic group, due to chronic insult from volume overload.^10^^,^^22^, ^23^, ^24^, ^25^

Reduced cytoplasmic reserve was appreciated in patients with severe PMR regardless of the presence of symptoms. This could signify myofibrillar loss and atrophy, indicating reduced reserve for contractility.^10^ In addition, vacuolization was frequently observed in patients with severe PMR regardless of presence of symptoms. The increased presence of autophagic vacuoles in patients with severe PMR regardless of symptoms could indicate cardiomyocyte autophagy from increased volume overload resulting in increased mechanical stretch, hypertrophy, or the development of heart failure.^10^^,^^26^^,^^27^ In fact, vacuoles and myofilament changes are commonly observed in patients with dilated cardiomyopathy and heart failure.^28^^,^^29^

Another ultrastructural alteration observed was mitochondrial derangement in the severe PMR group, regardless of presence of symptoms. Specifically, the linear registry of the mitochondrial arrangement along the sarcomeres was lost in the symptomatic group. Irregularities in the cristae as well as decreased cristae thicknesses were observed in both the symptomatic and asymptomatic groups. Furthermore, a significant decrease in mitochondria area as well as a decrease in the major-to-minor axis ratio of mitochondria are hallmark findings of mitochondrial dysfunction in heart failure and cardiomyopathy.^30^ These changes may contribute to the development of oxidative stress, which has been reported to be consistent with a response to volume overload.^10^^,^^31^ This chronic volume overload from severe PMR increases the pressure volume loop area, indicative of increased myocardial oxygen demand and consumption, which has been shown to activate oxidative stress-related genes.^32^ Studies have shown that in the settings of PMR, despite preserved ejection fraction, these adverse mitochondrial alterations could be a precursor to mitochondrial dysfunction and eventual LV dysfunction.^10^ In our observations, we identified an association between symptomatology and mitochondrial morphological changes in all patients with severe PMR. However, it remains unclear whether a causal relationship exists between symptoms and these mitochondrial alterations. Further careful investigation will be required to clarify this relationship.

There are a few limitations to this study, including limited samples available to obtain TEM images. Selection bias may exist in the control group. Additional testing such as immunohistochemistry testing may also be worthwhile to perform, but because of limited tissue size, priority was given to TEM analysis, and paired analysis was not feasible. We do intend to perform a larger cohort of studies to enroll more patients and investigate additional variables, such as LV strain and biomarkers associated with severe PMR with or without symptoms. We may also expand this study to further evaluate for any ultrastructural changes in patients with less-than-severe PMR. In addition, future studies in animals should be carried out to identify the point at which ultrastructural changes begin. Lastly, whether the ultrastructural changes observed in this study are reversible or whether disease progression can be halted after correction of PMR remains unknown. Addressing this question would require longitudinal follow-up studies, although conducting such studies in humans may pose ethical challenges. However, LV samples after successful MV repair operations may provide invaluable information regarding LV remodeling after surgical intervention. Future studies may be necessary to understand the impact of PMR correction and the timing of PMR correction on the underlying ultrastructural changes and cellular functions.

In conclusion, in patients with severe asymptomatic PMR and preserved LV function without atrial fibrillation, pulmonary hypertension, or significant LA enlargement, ultrastructural alterations of the LV myocardium are already evident and comparable with those observed in severe symptomatic PMR. These findings suggest that early surgical intervention in patients with severe PMR who are asymptomatic may help prevent further adverse remodeling and potentially improve clinical outcomes.

## Conflict of Interest Statement

The authors reported no conflicts of interest.

The *Journal* policy requires editors and reviewers to disclose conflicts of interest and to decline handling or reviewing manuscripts for which they may have a conflict of interest. The editors and reviewers of this article have no conflicts of interest.
